# Red to green: catching gene silencing as it takes hold

**DOI:** 10.1111/tpj.71188

**Published:** 2026-10-20

**Authors:** Charlay Wood

A green *Arabidopsis* seedling that ought to be magenta looks like a failed transformation. Xin Wei, Yanming Hou, Yuyang Si and colleagues have turned it into a screen. They gave the plant's silencing machinery a pigment gene to switch off and then looked for mutants that switched it back on. The screen recovered more than twice as many post‐transcriptional gene silencing (PTGS) alleles as any earlier reporter system has produced. Because the pigment can be scored on a living plant, from cotyledon to flower, it also shows when silencing takes hold, not just whether it does.

PTGS underpins antiviral immunity and endogenous gene regulation in plants (Hamilton & Baulcombe, 1999), and its core components came out of forward genetic screens using GFP, GUS or luciferase reporters (Dalmay et al., 2000; Mourrain et al., 2000). However, GFP requires excitation and imaging equipment; GUS assays require a substrate and destructive sampling; luciferase needs a substrate and a sensitive detector; and none are easy to analyse across thousands of seedlings. Earlier screens also produced more limited allelic series and made weak, developmentally changing phenotypes harder to resolve.

*RUBY* solves that a change in PTGS becomes a change in colour (He et al., 2020). However, a *35S::RUBY* plant is already red, and picking a slightly redder seedling out of thousands is no easy feat. Wei and colleagues therefore crossed a stable *35S::RUBY* line into *ein5‐1*, a mutant lacking the 5′→3′ exoribonuclease EIN5 (Olmedo et al., 2006), whose loss raises small RNA levels and strengthens silencing of coding genes (Wu et al., 2020; Zhang et al., 2015). In this background, the reporter's own aberrant transcripts trigger silencing of *RUBY* itself. Plants accumulate *RUBY*‐derived 21‐ and 22‐nt siRNAs and are fully green as early as 4–5 days after germination, early enough to screen seedlings by eye. Green is now the starting point, and a known *rdr6‐11* mutation turns the pigment back on (Figure 1a). Losing colour means silencing is working; regaining it means silencing has failed.

**Figure 1:**
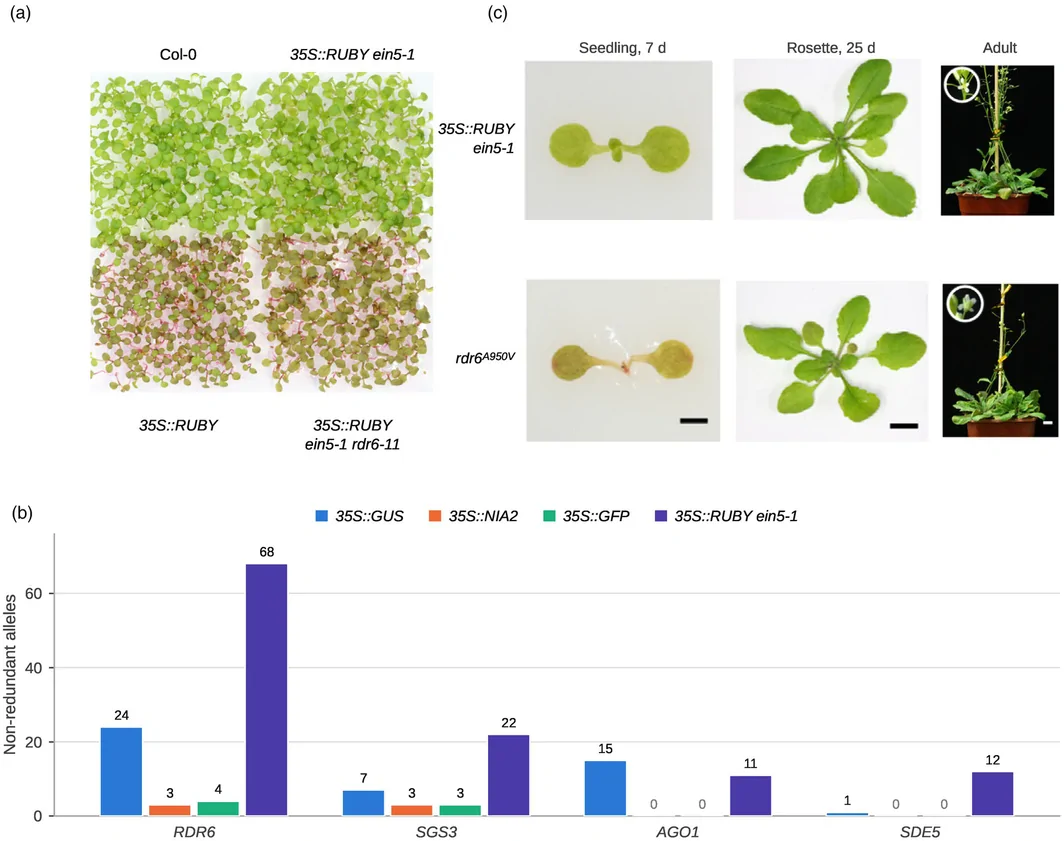
Silencing made visible in the *35S::RUBY ein5‐1* screen. (a) The screening logic. Ten‐day‐old seedlings of Col‐0, *35S::RUBY*, *35S::RUBY ein5‐1* and *35S::RUBY ein5‐1 rdr6‐11*. Betacyanins accumulate when *RUBY* is expressed, are lost when *ein5‐1* intensifies PTGS and return when RDR6 function is removed, so restored pigment accumulation reports weakened or failed silencing. (b) Scale of the allelic series. Non‐redundant alleles recovered for *RDR6*, *SGS3*, *AGO1* and *SDE5* in the *35S::RUBY ein5‐1* screen, set against the *35S::GUS*, *35S::NIA2* and *35S::GFP* systems, all counted under the authors' unified scheme. (c) Silencing established over developmental time. Seven‐day‐old seedlings, 25‐day‐old rosettes and adult plants of the weak allele *rdr6* ^A950V^ besides the *35S::RUBY ein5‐1* parent, showing the red‐to‐green transition. (a, c) Adapted from figures 1(a) and 3(a) of Wei et al. (2026); (b) replotted from table 1 of Wei et al. (2026).

EMS mutagenesis and bulked segregant resequencing identified 162 non‐redundant alleles. Most lie in the four core PTGS components: 68 in *RDR6*, 22 in *SGS3*, 11 in *AGO1* and 12 in *SDE5*, with 49 more spread across other loci. The latter represent candidate regulators distributed across 26 genes and remain to be functionally validated. The classic *35S::GUS* system has produced 61 alleles across several studies on the same count, including a single *sde5* allele against 12 identified by Wei et al. (Figure 1b). Every allele here was recovered because silencing was weakened, delayed or lost. The same logic sets this *RUBY*‐based screen's limitation: with no pigment left to lose in the green parental line, it cannot report a mutation that strengthens silencing. Finding such mutants would require a complementary screen, starting from a red plant and looking for loss of pigment.

Scoring the same plant as a seedling, as a rosette and as an adult meant the screen could show not just whether silencing failed, but when. *rdr6* ^A950V^ is the clearest case. Pigment is visible in its seven‐day‐old seedlings but gone by the 25‐day rosette and the adult (Figure 1c), and by 25 days its *RUBY*‐derived siRNAs have climbed close to the levels in the 35S::RUBY ein5‐1 parent. The authors interpret this as a partial loss allele that delays RDR6‐dependent silencing rather than blocking steady‐state siRNA production, and propose that siRNAs gradually accumulate until silencing is complete. PTGS, in Wei Yan's words, ‘is not simply an on/off switch, but rather a process that is progressively established over time’.

Partial loss alleles are also what make structure–function work possible, and RDR6 shows why. Mapped onto predicted structures, the missense changes are not randomly distributed. They concentrate at the polymerase active site, at the N‐terminal RNA‐binding module, and around the linkers at the junctions where domains meet. The N‐terminal module is where RDR6 contacts its partner SGS3, and all three substitutions tested there, G140R, D245N and R275W, weakened the interaction in bimolecular fluorescence complementation assays. AGO1, the protein that carries the small RNA and cuts its target, shows why partial alleles are especially valuable: canonical loss‐of‐function mutations frequently cause lethality or severe developmental defects, and this screen recovered 11 viable alleles, all missense.

The recovered *sde5* alleles point somewhere else entirely, to transcript structure rather than protein structure. SDE5 works with SGS3 to recruit RDR6 (Yoshikawa et al., 2021), and one of its alleles, W93*, is a nonsense mutation at codon 93 that should therefore be a null. It is not. The authors found that *SDE5* is transcribed from two start sites and that W93* reduces the amount of the scarce long transcript without changing the level of the abundant short one. This allele supports the conclusion that the long transcript, for all its scarcity, is needed for full SDE5 function.

Earlier screens established whether a gene was needed for silencing. This one extends that work by asking how much each contributes, and when, on living plants, by eye and across developmental stages. Some of the mutations map to genes with no known connection to silencing, although these candidate regulators still require validation. There is clearly more of this pathway to find, and now there is a simple way to go looking.

## Data Availability

Data sharing not applicable to this article as no datasets were generated or analysed during the current study.
